# Quantitative analysis of structural abnormality of the vestibular system in progressive and non-progressive adolescent idiopathic scoliosis (AIS) using MRI techniques

**DOI:** 10.1186/1748-7161-10-S1-O50

**Published:** 2015-01-19

**Authors:** Winnie CW Chu, Lin Shi, Defeng Wang, Yong Qiu, Jack CY Cheng

**Affiliations:** 1 Department of Imaging and Interventional Radiology, The Chinese University of Hong Kong, Hong Kong; 2 Department of Medicine and Therapeutics, The Chinese University of Hong Kong, Hong Kong; 3 Spine Surgery, The Affiliated Drum Tower Hospital of Nanjing University Medical School, Nanjing, China; 4 Department of Orthopaedics and Traumatology, The Chinese University of Hong Kong, Hong Kong; 5 Joint Scoliosis Research Center of the Chinese University of Hong Kong and Nanjing University, China

## Introduction

Previous studies suggested that asymmetric growth of vestibular system (VS) is related to the development of AIS. It provided a possible direction to resolve the unclear etiopathology of AIS and to reveal the difference in vestibular system morphology between progressive and non-progressive AIS patients.

## Objectives

The goal of this study is to investigate the difference between the progressive and non-progressive AIS patient as compared with age-matched normal controls using computational morphometric approaches on MR images.

## Materials and methods

Twenty-five non-progressive (NP-AIS) and 17 progressive (P-AIS) right-thoracic AIS patients and 20 age-matched normal controls (NC) were recruited in this research. High resolution T2-weighted images of their VS were obtained. The anatomical variances of the semicircular canals were described using approximated best-fit circle. Lengths and angles of the lines joining centers of the best-fit circles, radius of the circles and the rotational angles around the three major orthogonal axes were used for shape description of the VS. The measurements of the AIS groups were compared with those of the normal control group using one-way ANOVA.

## Results

Statistically significant differences were observed in the shape analysis of the left-side VS between AIS and normal controls. Firstly, the distance between the centers of the lateral and superior canals was P-AIS<NP-AIS<NC (p=0.0370). Secondly, the angle between the center-joining lines at the posterior canal was P-AIS<NP-AIS<NC (p=0.0018). In the right-side VS, no significant group difference was detected.

## Conclusion and significance

Abnormalities of VS structure affect the postural balance, vestibular and proprioceptive functions. Our results preliminarily confirmed the shape deviations in AIS and the difference is more prominent in the progressive group than the non-progressive group. A longitudinal study will be conducted to establish the association of vestibular dysfunction with curve progression of spinal deformation of AIS.

